# Supplementary figures and images for: Senescent Cells in Growing Tumors: Population Dynamics and Cancer Stem Cells
Source: PLoS Comput Biol. 2012 Jan 19;8(1):e1002316. doi: 10.1371/journal.pcbi.1002316 (PMC3261911; doi:10.1371/journal.pcbi.1002316)

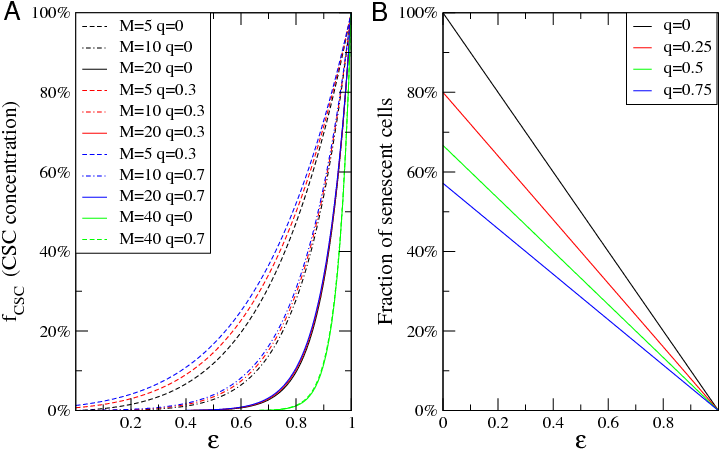

Supplement: Figure S1 — Asymptotic fractions of CSCs and senescent cells in the model. The asymptotic fraction of CSCs (A) and senescent cells (B) as a function of the proliferation parameter and for different values of the number of duplications needed by cancer cells to become senescent and of the probability of cell death . Unlike the fraction of CSC, the fraction of senescent cells does not depend on . (TIFF) [file pcbi.1002316.s001.tif]

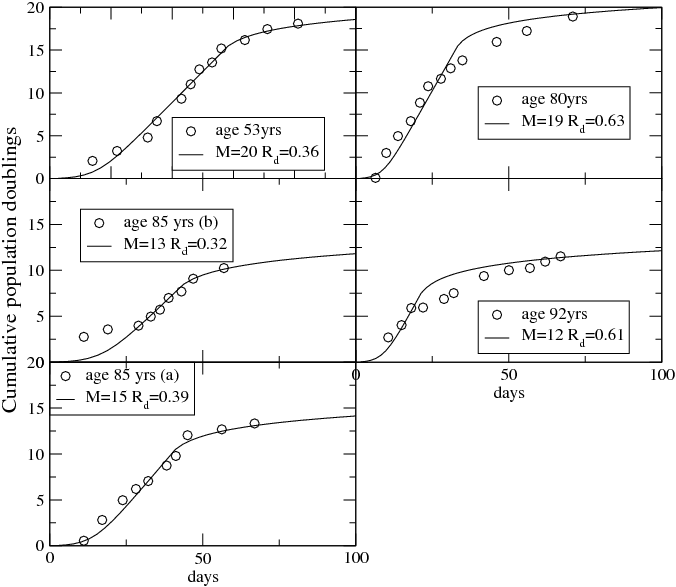

Supplement: Figure S2 — Growth curves of mesenchymal stem cells (HIP). The growth of populations of MSC isolated from the bone marrow from the femoral hip in terms of cumulative population doublings are fitted by the model. Experimental data are obtained from Ref. [29]. Cell populations refer to donors with different ages. The best fit is obtained varying and in Eq. 7. (TIFF) [file pcbi.1002316.s002.tif]

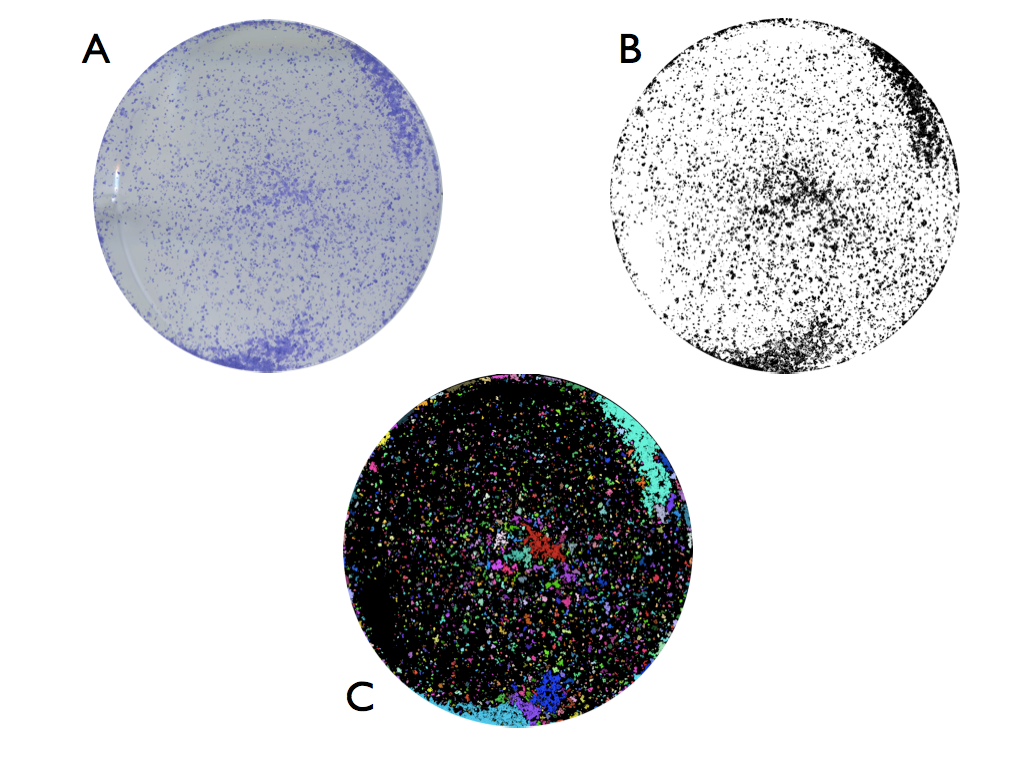

Supplement: Figure S4 — Cluster analysis of crystal violet assay. A) To analyze the crystal violet assay, we first photograph the multiwell and isolate a single well. B) Using the image analysis software Gimp we select by color the spots, eliminate the background and threshold the remaining spots in order to obtain a two color image. C) We apply the Hoshen-Kopelman cluster algorithm to identify individual colonies, recolored here with random colors for visualization purposes. (TIFF) [file pcbi.1002316.s004.tif]

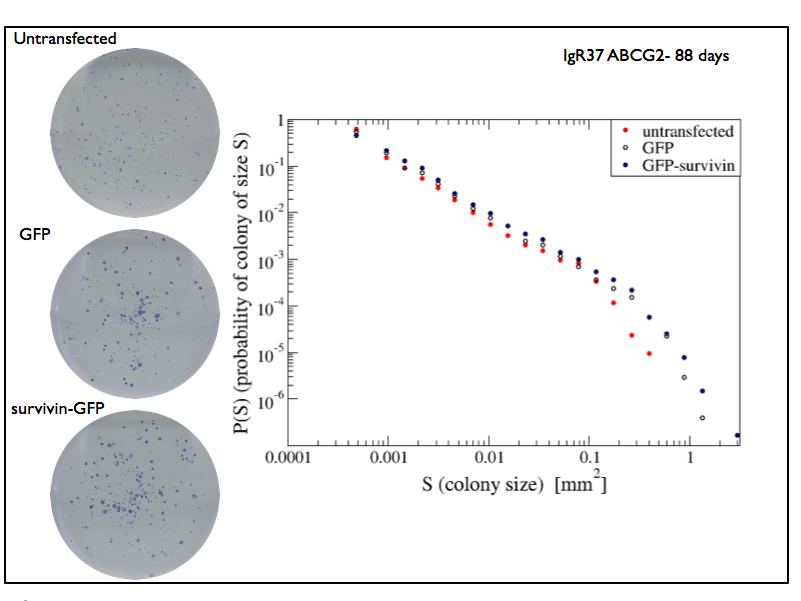

Supplement: Figure S5 — Effect of survivin on senescence away from the peak of . we show the distribution of colony sizes obtained from crystal violet assay for untransfected, GFP and GFP-survivin cells. In this experiment 500 cells were plated after 88 days of cultivation (see Table S2). We see a small effect due to GFP and survivin. (TIFF) [file pcbi.1002316.s005.tif]
