# Supplementary material for: Senescent Cells in Growing Tumors: Population Dynamics and Cancer Stem Cells
Source: PLoS Comput Biol. 2012 Jan 19;8(1):e1002316. doi: 10.1371/journal.pcbi.1002316 (PMC3261911; doi:10.1371/journal.pcbi.1002316)
Supplement: Table S1 — Evolution of senescence marker. The evolution of the percentage of positive cells for ABCG2 sorted IGR39 and IGR37 cell populations. (PDF) [file pcbi.1002316.s006.pdf]

IGR39 ABCG2+		IGR39 ABCG2-		IGR37 ABCG2+		IGR37 ABCG2-	
day	%β-gal	day	%β-gal	day	%β-gal	day	%β-gal
25	1.7 ± 0.5	24	2.5 ± 0.7	1	1.0 ± 0.2	1	1.0 ± 0.5
79	8 ± 1	43	2.4 ± 0.5	25	0.4 ± 0.1	56	3.6 ± 0.5
89	48 ± 5	75	9.4 ± 0.6	81	23 ± 2	80	11 ± 3
96	47 ± 4	80	37 ± 6	88	23 ± 2	88	36.0 ± 3
103	32 ± 4	90	92 ± 8	102	14 ± 2	98	66 ± 4
111	$29 \pm 2.$	97	87 ± 8	110	11 ± 2	111	36 ± 6
121	14 ± 5	104	89 ± 7	120	1.30 ± 0.05	119	11 ± 1
		112	66 ± 5			129	0.60 ± 0.05
		122	14 ± 4				
