# Supplementary material for: Senescent Cells in Growing Tumors: Population Dynamics and Cancer Stem Cells
Source: PLoS Comput Biol. 2012 Jan 19;8(1):e1002316. doi: 10.1371/journal.pcbi.1002316 (PMC3261911; doi:10.1371/journal.pcbi.1002316)
Supplement: Table S2 — Crystal violet assays. A summary of the results obtained with the crystal violet assay for GFP and GFP-survivin transfected cells at different stages of the growth. We report the total number of colonies and the fraction of area covered by colonies. (PDF) [file pcbi.1002316.s007.pdf]

Experiment	cells plated	n. of colonies	area covered [%]
ABCG2- GFP (98 days)	200	3987 ± 1521	2.25 ± 0.96
ABCG2- GFP-survivin (98 days)	200	7320 ± 1560	25.4 ± 7.5
unsorted GFP (start)	200	1514 ± 141	2.6 ± 0.2
unsorted GFP-survivin (start)	200	1161 ± 78	1.8 ± 0.1
ABCG2- GFP (88 days)	500	640 ± 86	1.8 ± 0.3
ABCG2- GFP-survivin (88 days)	500	750 ± 50	2.6 ± 0.2
ABCG2- untransfected (88 days)	500	1201 ± 202	1.7 ± 0.2
