# Supplementary material for: Senescent Cells in Growing Tumors: Population Dynamics and Cancer Stem Cells
Source: PLoS Comput Biol. 2012 Jan 19;8(1):e1002316. doi: 10.1371/journal.pcbi.1002316 (PMC3261911; doi:10.1371/journal.pcbi.1002316)
Supplement: Table S3 — Fit covariance matrix. The covariance matrix and the t-statistics of the joint fit of the growth curves and concentration with the CSC model. (PDF) [file pcbi.1002316.s008.pdf]

	q	ϵ	M	$c^{(+)}$	$c^{(-)}$	R_d
q	1.48e-03	-6.90e-05	1.62e-03	1.16e-03	8.93e-05	1.04e-05
ϵ	-6.90e-05	1.17e-04	3.17e-05	1.99e-03	4.75e-05	-9.23e-07
M	1.62e-03	3.17e-05	1.26e-02	1.91e-02	7.38e-04	4.58e-05
$c^{(+)}$	1.16e-03	1.99e-03	1.91e-02	1.22e-01	3.75e-03	2.28e-04
$c^{(-)}$	8.93e-05	4.75e-05	7.38e-04	3.75e-03	1.75e-04	9.48e-06
R_d	1.04e-05	-9.23e-07	4.58e-05	2.28e-04	9.48e-06	8.55e-07
t-statistic	9.9	65.4	334.9	11.6	10.0	414.9
